# Endoscopic ultrasound‑guided stricturotomy for complete anastomotic occlusion following Hartmann’s reversal

**DOI:** 10.1055/a-2849-6115

**Published:** 2026-05-05

**Authors:** Qinghua Zhong, Xiaochuang Feng, Dezheng Lin, Juan Li, Mingli Su, Yongcheng Chen, Xuefeng Guo

**Affiliations:** 1 Department of General Surgery (Endoscopic Surgery) 373651 Sun Yat-sen University Sixth Affiliated HospitalGuangzhouChina; 2 Guangdong Provincial Key Laboratory of Colorectal and Pelvic Floor Diseases 373651 Sun Yat-sen University Sixth Affiliated HospitalGuangzhouChina; 3 Biomedical Innovation Center 373651 Sun Yat-sen University Sixth Affiliated HospitalGuangzhouChina; 4 Department of General Surgery (Colorectal surgery) 373651 Sun Yat-sen University Sixth Affiliated HospitalGuangzhouChina

Complete anastomotic occlusion after colorectal surgery is rare but challenging, traditionally necessitating reoperation 1 . Although therapeutic endoscopy has been reported, it typically relies on endoscopic rendezvous techniques, which are inapplicable without a proximal stoma 2 . We herein report a case of complete anastomotic occlusion post-Hartmann’s reversal successfully treated with endoscopic ultrasound (EUS)‑guided stricturotomy.

**Fig. 1:**
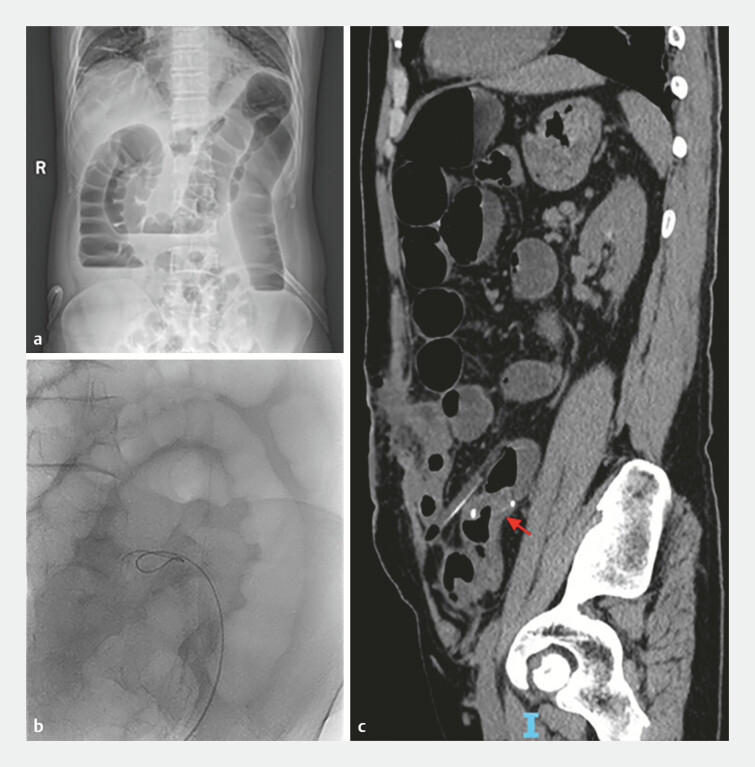
Evaluation prior to endoscopic ultrasound‑guided stricturotomy. **a** An upright abdominal radiograph showing dilated bowel loops suggestive of obstruction. **b** CT showing an anastomotic stricture (red arrow). **c** Contrast study demonstrating complete anastomotic occlusion with failed guidewire passage. CT, computed tomography.

A 67-year-old man, 5 months after laparoscopic sigmoidectomy with Hartmann’s procedure for obstructive sigmoid colon cancer (pT3N0), underwent Hartmann’s reversal at our hospital. On postoperative day 4, he presented with abdominal pain ( Fig. 1 **a** ). Computed tomography revealed anastomotic inflammatory edema causing near-total proximal colonic obstruction ( Fig. 1 **b** ). Contrast study demonstrated complete anastomotic occlusion ( Fig. 1 **c** ).

**Fig. 2:**
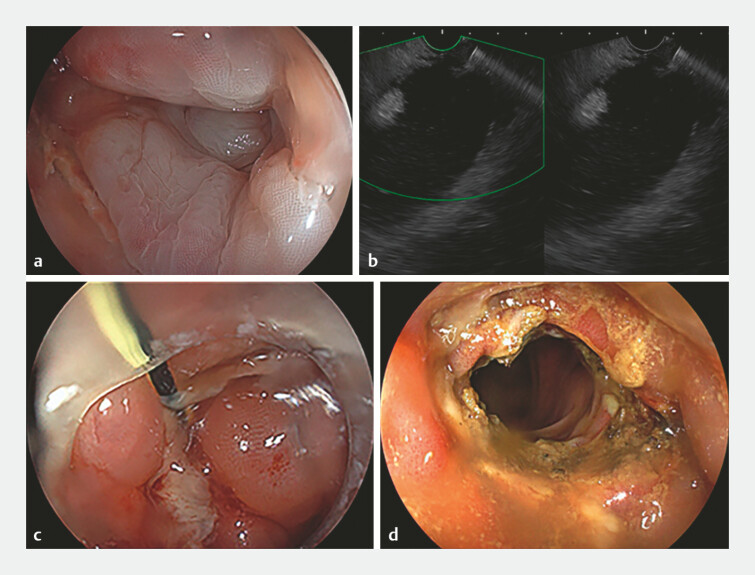
An EUS‑guided stricturotomy procedure. **a** Colonoscopy confirming complete occlusion at the colo-colonic anastomosis. **b** EUS showing guidewire advancement across the occlusion into the proximal bowel via puncture needle. **c** Colonoscopy demonstrating guidewire traversal through the anastomotic site. **d** A post-procedural view showing successful anastomotic recanalization. EUS, endoscopic ultrasound.

**Fig. 3:**
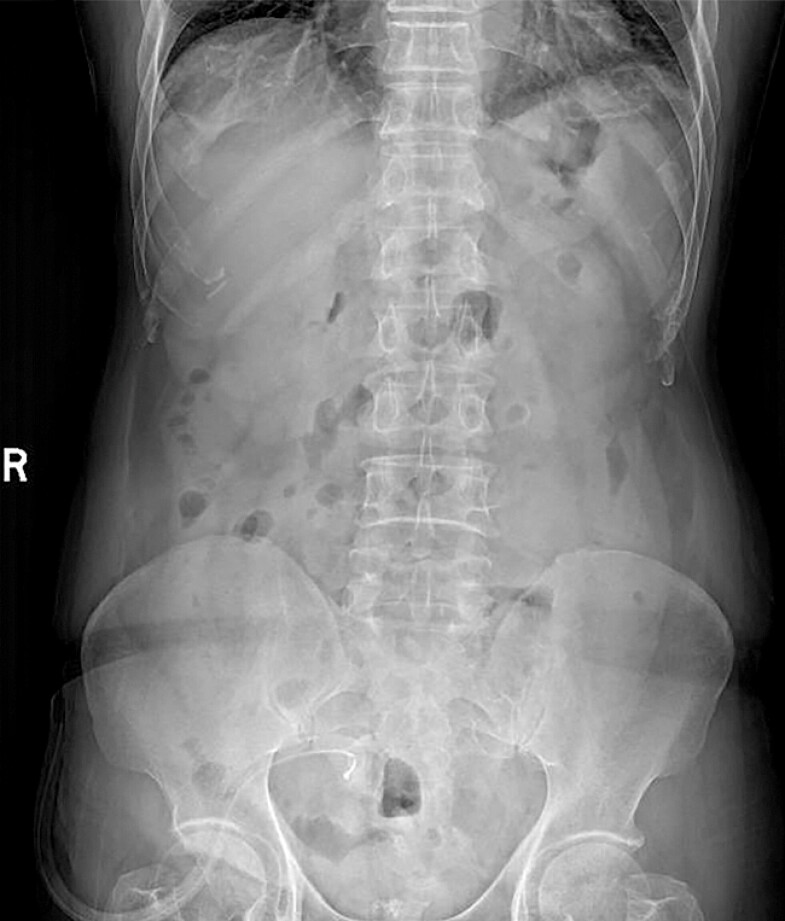
An unremarkable abdominal radiograph after stricturotomy.

We opted to perform EUS-guided stricturotomy. Initial colonoscopy confirmed complete colo-colonic anastomotic occlusion ( Fig. 2 **a** and Video 1 ). A linear echoendoscope was advanced to the obstruction’s anal side, with EUS demonstrating a dilated proximal bowel loop filled with fluid and particulate matter. After Doppler imaging confirmed no intervening vessels, the occluded anastomosis was punctured with a 19-gauge needle. Aspiration of the copious fecal material verified successful proximal bowel lumen access, and a 0.035-inch guidewire was inserted through the needle ( Fig. 2 **b** ). Under wire guidance, a hook knife incised the stricture, achieving successful anastomotic recanalization with massive fecal evacuation ( Fig. 2 **c** ). The colonoscope was advanced unimpeded through the patent anastomosis into the proximal colon ( Fig. 2 **d** ). To ensure the correct direction and avoid perforation, the puncture site was set at the anastomotic midpoint, determined via EUS according to the anal and proximal bowel orientation, and incision was kept aligned with the guidewire. Post-procedure, the patient’s abdominal pain resolved completely. Upright abdominal radiography showed no obstruction or pneumoperitoneum ( Fig. 3 ). He resumed normal diet and bowel function and was discharged in a stable condition.

Management of complete anastomotic occlusion after Hartmann’s reversal using EUS-guided stricturotomy. EUS, endoscopic ultrasound.Video 1

EUS‑guided stricturotomy may serve as a valuable therapeutic alternative for complete anastomotic strictures in which endoscopic rendezvous techniques are not applicable.

Endoscopy_UCTN_Code_TTT_1AS_2AG
